# Structural neuroimaging phenotypes and associated molecular and genomic underpinnings in autism: a review

**DOI:** 10.3389/fnins.2023.1172779

**Published:** 2023-06-30

**Authors:** Charlotte M. Pretzsch, Christine Ecker

**Affiliations:** ^1^Department of Forensic and Neurodevelopmental Sciences, Institute of Psychiatry, Psychology, and Neuroscience, King’s College London, London, United Kingdom; ^2^Department of Child and Adolescent Psychiatry, Psychosomatics and Psychotherapy, University Hospital Frankfurt, Goethe University, Frankfurt, Germany

**Keywords:** autism, autism spectrum disorder, neuroanatomy, cortical thickness, surface area, cortical volume, gyrification, grey-white matter tissue contrast

## Abstract

Autism has been associated with differences in the developmental trajectories of multiple neuroanatomical features, including cortical thickness, surface area, cortical volume, measures of gyrification, and the gray-white matter tissue contrast. These neuroimaging features have been proposed as intermediate phenotypes on the gradient from genomic variation to behavioral symptoms. Hence, examining what these proxy markers represent, i.e., disentangling their associated molecular and genomic underpinnings, could provide crucial insights into the etiology and pathophysiology of autism. In line with this, an increasing number of studies are exploring the association between neuroanatomical, cellular/molecular, and (epi)genetic variation in autism, both indirectly and directly *in vivo* and across age. In this review, we aim to summarize the existing literature in autism (and neurotypicals) to chart a putative pathway from (i) imaging-derived neuroanatomical cortical phenotypes to (ii) underlying (neuropathological) biological processes, and (iii) associated genomic variation.

## 1. Introduction

Autism spectrum disorder (ASD; henceforth referred to as autism) is a lifelong, complex, multifactorial, neuropsychiatric condition reported to occur in 1 out of 54 individuals (Knopf, 2020). Core features include social communication difficulties and restricted and repetitive patterns of interests and behaviors (American Psychiatric Association, 2013). These symptoms are accompanied by multifaceted differences in brain structure. For instance, structural magnetic resonance imaging (sMRI) studies have identified differences in (the developmental trajectories of) multiple neuroanatomical cortical features. Among these are the commonly implicated cortical thickness, surface area, cortical volume, measures of gyrification, and the grey-white matter tissue contrast (GWC) (Pretzsch and Ecker, 2022). Combined, these neuroimaging features have been proposed as intermediate phenotypes on the gradient from genomic variation to behavioral symptoms (Carter et al., 2017). Therefore, examining what these cortical proxy markers represent, i.e., disentangling their associated molecular and genomic underpinnings, could provide crucial insights into the etiology and pathophysiology of autism.

Previous studies suggest that inter-individual neuroanatomical variability at the macroscopic level may be influenced by microscopic variability. For example, postmortem studies have linked neuroanatomical differences to minicolumnar pathology, abnormal neuronal numbers and sizes etc [reviewed e.g., in Fetit et al., 2021]. This microscopic variability in turn is thought to result from a complex set of cellular/molecular processes rooted in genetic, epigenetic, and environmental features. In line with this, genetic studies in autism to date have implicated more than 1,000 genetic variants in the etiology of autism (O’Roak et al., 2012). Notably, the relationships between neuroanatomical variability and underlying neurobiological mechanisms and genomic features are complex and subject to significant heterogeneity. Nonetheless, an increasing number of studies are exploring these associations both indirectly and directly *in vivo*, across age, and for different neuroanatomical features.

Combined, this research has given rise to a wealth of publications. However, to our knowledge, no study or review article has yet attempted to integrate the information provided by these studies on typical and atypical neurodevelopment to chart a putative pathway from (1) commonly examined neuroanatomical imaging cortical phenotypes to (2) molecular mechanisms and (3) associated genomic variation. Hence, this is the aim of the present review. Note that here we focus on research in humans [reviews in mice: (Collins et al., 2019; Wang et al., 2020); reviews in zebrafish: (Anand and Mondal, 2020; Jenett, 2020)]. Also, we would like to highlight that this is not a review of imaging genetics studies *per se*, but a review of studies examining the biological associates of imaging markers (which include imaging genetics studies). Further, while there is a growing body of literature addressing the cerebellum in neurotypical and atypical individuals, the present review focuses on the cerebral cortex; for additional information concerning the cerebellum, we refer the reader to e.g (Fatemi et al., 2012; Hampson and Blatt, 2015; De Zeeuw et al., 2021). Finally, we recognize the crucial role of biological sex differences in genotypes, phenotypes (including neuroanatomy), and the genetic regulation of phenotypes, in both the neurotypical and autistic brain. For example, we recently reported an overlap between those neuroanatomical features characteristic of neurotypical males (vs. females) with those of autistic individuals (Floris et al., 2023). In autistic females, this male-shifted neuroanatomy was associated with gene expression patterns of midgestational cell-types (Floris et al., 2023). Taken together, these findings illustrate the complexity of sex differences in neuroimaging phenotypes and associated molecular mechanisms and genomic variation in the autistic and neurotypical brain. However, a discussion of these sex differences is beyond the scope of this review; hence, we refer the reader to previous work on this topic (e.g., Lai et al., 2015; Chen and Van Horn, 2017; Cauvet et al., 2019).

Hence, in this review, we will first outline different analytical techniques applied to sMRI data to derive commonly studied neuroanatomical features, including cortical thickness, surface area, cortical volume, cortical folding/gyrification, and the GWC. We will briefly discuss each feature and its development across age in neurotypicals and in autism. Second, we will introduce methods commonly used to examine the biological processes thought to contribute to neuroanatomy. We will provide a brief overview over shared and distinct developmental pathways between features and detail the potential microscopic underpinnings of each feature in neurotypicals and in autistic individuals. Third, we will summarize efforts to examine the genomic associates of these microscopic processes and macroscopic features. We will provide a brief overview over commonly used genomic analytic approaches. We will then outline the genomic features related to (atypical) cortical development and neuroanatomy in general, and each individual cortical feature, in neurotypicals and in autism. Fourth, we will discuss current and future research trends, including those based on existing limitations and the potential of large-scale collaborations. Fifth, we will conclude our review with a brief summary.

## 2. Neuroimaging features

The current gold standard for investigating neuroanatomy *in vivo* is sMRI, which includes T1-weighted imaging (high-level structural detail) and diffusion tensor imaging (DTI; captures white matter organization) (Schumann and Nordahl, 2011). This review will focus on T1-weighted imaging; for additional information concerning DTI, we refer the reader to e.g (Ranzenberger and Snyder, 2023). Similarly, a discussion of alternative neuroimaging modalities, including methods used to assess brain function (e.g., functional MRI) and brain biochemistry (e.g., magnetic resonance spectroscopy) is beyond the scope of this review, which centers on structural neuroimaging. Broadly, sMRI leverages the magnetic properties of different tissue types (e.g., gray and white matter) to produce high-resolution images of the brain. Traditional sMRI studies have explored neuroanatomy (based on cortical or regional brain volume) using time- and labor-intensive manual segmentation approaches, such as hand-tracing of individual pre-defined regions (e.g., Allen et al., 2002). To overcome these challenges, increasing efforts have been directed toward developing automated segmentation methods, including e.g., deep-learning approaches [reviewed in Fawzi et al. (2021)]. Also, advances in spatially unbiased computational techniques, e.g., voxel-based morphometry (VBM), have enabled the simultaneous volumetric examination of multiple (sub)cortical structures based on local concentrations of gray matter (Wright et al., 1995). Currently used techniques, such as surface-based morphometry (SBM) (MacDonald et al., 2000), now allow us to further disentangle the complex morphometric constituents underpinning cortical volume (i.e., cortical thickness and surface area); and to explore other features of neuroanatomy, including measures of curvature and the GWC (Pretzsch et al., 2019; Pretzsch and Ecker, 2022). In the following, we will briefly describe (1) how each of these morphometric measures is identified, (2) the (neuro)typical trajectory of different features, and (3) how−and where−individual features (and their developmental trajectory) differ in autism. Please note that, while our review focuses on a selection of commonly studied features, research on additional features is rapidly expanding. For instance, a recent study identified more extra-axial cerebrospinal fluid in autistic compared to neurotypical children, which may represent a potential early stratification biomarker (Shen et al., 2018).

### 2.1. Cortical thickness

**FIGURE 1:**
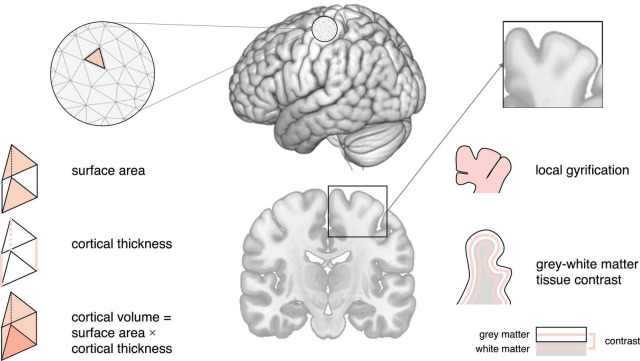
Schematic of neuroimaging features mentioned in this review.

**FIGURE 2:**
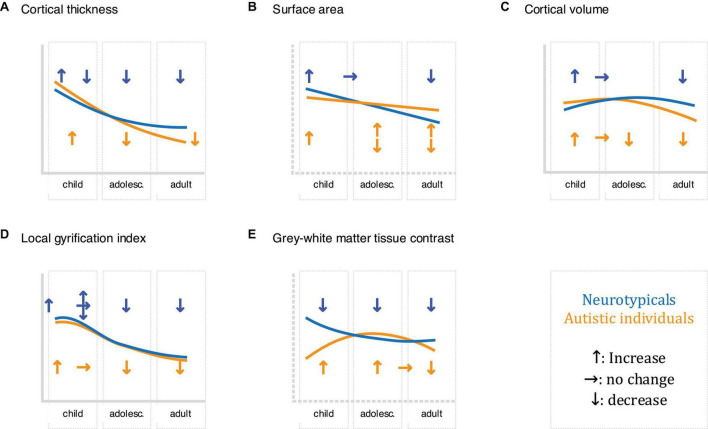
Schematic of the development of the described neuroimaging features (**A**: cortical thickness, **B**: surface area, **C**: cortical volume, **D**: local gyrification index, and **E**: grey-white matter tissue contrast) in childhood, adolescence, and adults in neurotypicals (shown in blue) and autistic individuals (displayed in orange), where upward arrows indicate increases, horizontal arrows indicate stagnation/plateau, and downward arrows indicate decreases. Where research has been inconclusive/contradicting, multiple arrows are presented. Please note that this schematic is not comprehensive: different growth trajectories have been reported both within studies (e.g., for different brain regions) and between studies. For more information concerning neuroanatomical differences and their trajectories in autism, see e.g (Courchesne et al., 2007; Ecker et al., 2014, 2015; Zielinski et al., 2014; Lange et al., 2015; Mensen et al., 2016; Li et al., 2021).

Cortical thickness is commonly defined as the closest distance from the gray matter/white matter boundary (inner gray matter surface) to the gray matter/cerebrospinal fluid (CSF) boundary (outer gray matter surface) (Fischl et al., 1999). In neurotypicals, cortical thickness is thought to increase and peak early in life, followed by a rapid decrease during childhood and adolescence, and a decelerated decrease from adulthood onward (Huttenlocher, 1979; Fjell et al., 2015). In autism, large-scale studies have reported increased cortical thickness in frontal, temporal, parietal, and occipital regions from childhood onward, with differences diminishing during adulthood (Khundrakpam et al., 2017; Bedford et al., 2020). Also, autism has been associated with an accelerated thinning of the cortical mantle during adolescence and adulthood, particularly in the frontal, parietal, and occipital lobe (less in the temporal lobe) (Zielinski et al., 2014; Wallace et al., 2015). Combined, these studies point to region-dependent differences in cortical thickness, not only cross-sectionally, but also in terms of the developmental trajectory of cortical thickness in autism. Figures 1, 2A.

### 2.2. Surface area

Surface area is typically quantified as the area of the cortex at a given point on the cortical surface (i.e., the sum of faces in the polygon mesh representation of the cortex at a particular vertex) (Winkler et al., 2012). In neurotypicals, surface area increases rapidly during early childhood (Lyall et al., 2015), with a peak in late childhood/adolescence (Raznahan et al., 2011b), and a decline throughout adulthood (Hogstrom et al., 2013). Previous studies show that surface area develops atypically in autism, with studies in young children reporting early overgrowth (resulting in an enlargement of the cortex) before the age of 2 years in temporal, frontal, parietal, and occipital regions (Hazlett et al., 2011). Following this, some studies have reported reduced surface area in childhood, followed by an atypically steep increase throughout adulthood (Ecker et al., 2014); while others reported reduced surface area in childhood followed by an atypically low decline, resulting in increased surface area in adulthood (Mensen et al., 2016). These discrepancies may be due to methodological considerations (e.g., investigated regions) and the large phenotypic heterogeneity that is characteristic of autism. Regardless, they point to regional differences in surface area in autism, which may further vary across age. Figures 1, 2B.

### 2.3. Cortical volume

Cortical volume is the product of cortical thickness and surface area. In neurotypicals, the development of cortical volume is thought to follow an inverted U-shape, peaking between ∼7–10 years of age (Raznahan et al., 2011b). Notably, cortical thickness and surface area contribute differently to cortical volume, although in neurotypical children, adolescents, and adults, (change in) cortical volume is thought to be driven predominantly by (change in) cortical thickness (Storsve et al., 2014; Ducharme et al., 2015). In autism, cortical volume is significantly increased during childhood, followed by a plateau during later childhood (∼8–9 years of age), and an accelerated decline from adolescence onward (Courchesne et al., 2011a). Moreover, the contributions of cortical thickness and surface area to cortical volume may differ in autism. More specifically, in autistic adults, spatial patterns of differences in cortical thickness and surface area were largely non-overlapping, i.e., the probability that any one point on the cortex displayed a difference in both cortical thickness and surface area was very low (Ecker et al., 2013). Given the distinct developmental trajectories of cortical thickness and surface area across age (Hazlett et al., 2011; Ecker et al., 2014; Wallace et al., 2015), it is possible that early (< 2 years) differences in cortical volume are driven by atypical surface area, while later (childhood/adolescence) differences are linked to atypical cortical thickness and/or surface area. Nonetheless, additional research is required to confirm this. Figures 1, 2C.

### 2.4. Gyrification

The local gyrification index (lGI) quantifies the ratio of inner sulcal folds compared to the outer smooth cortical surface (a larger lGI indicates greater cortical folding) (Schaer et al., 2012). In neurotypicals, studies examining the developmental trajectory of local gyrification have reported inconsistent results, with some studies showing a rapid increase during fetal development (White et al., 2010), an increase/stagnation/decrease in childhood (White et al., 2010; Libero et al., 2019), and a decrease in adolescence and adulthood (Klein et al., 2014). In autism, a longitudinal study in children reported lower lGI in the fusiform gyrus at 3 years of age (Libero et al., 2019). Moreover, unlike neurotypicals who displayed a stable/decreasing lGI, autistic children showed an increase in lGI in frontal, temporal, and parietal regions; and a stable lGI in the occipital lobe between the ages of 3 and 5 years of age (Libero et al., 2019). Further, studies in autistic adolescents have reported increased lGI in frontal, temporal, and parietal regions; as well as an accelerated decline in lGI in frontal, precentral, and occipital regions (Kohli et al., 2019a). Last, studies have reported widespread reductions in lGI in autistic adults (Kohli et al., 2019b). Taken together, these studies suggest that autism is associated with regionally specific differences in cortical gyrification that also vary across age. Figures 1, 2D.

### 2.5. Gray-white matter tissue contrast

The GWC characterizes difference in signal intensities of gray and white matter tissue measured using *in vivo* sMRI. It is frequently used to identify the gray/white matter boundary and is also relevant to other neuroanatomical imaging markers (e.g., surface area) that rely on the correct placement (i.e., identification) of the lower (i.e., white matter) boundary of the cortex. Notably, the GWC has both methodological [e.g., the variability in field strength, pulse sequence, and data processing parameters (Han et al., 2006)], and biological [e.g., variability across age, regions, and medical conditions (Panizzon et al., 2012)] origins. In neurotypicals, the GWC is reported to show regionally specific decreases. These decreases begin in childhood, are strongest in adolescence, and decelerate in adulthood (Mann et al., 2018). Longitudinal studies in toddlers who later developed autism have reported widespread increases in GWC in frontal, temporal, and parietal regions, where an earlier onset of autism symptoms was associated with slower GWC rates of change (Godel et al., 2021). In autistic adolescents and adults, the GWC was reported to follow an inverted U-shape with a peak in late adolescence (Mann et al., 2018). Thus, region- and age-dependent differences in GWC may be a characteristic hallmark of atypical brain development in autism. Figures 1, 2E.

Taken together, previous cross-sectional and longitudinal studies have examined neuroanatomical features in both the neurotypical and atypical (autistic) brain; and identified age- and region-specific variation in several of these features in autism. In the following section, we will detail the potential neurobiological and/or histological correlates of these neuroanatomical features.

## 3. Neurobiological underpinnings of different neuroanatomical features

Commonly used approaches to study (neuropathological) mechanisms in humans are largely based on the analysis of postmortem brain tissue (for a review, see Fetit et al., 2021). Among many others, these techniques include (1) microscopic histology [e.g., used to determine cell numbers, types, and sizes (Courchesne et al., 2011b)]; (2) *in situ* hybridization [e.g., used to determine enzyme levels (Yip et al., 2009)]; (3) metabolomics [e.g., used to probe metabolic pathways (Kurochkin et al., 2019)]; and (4) microanalysis and western blotting [e.g., used to examine receptor densities (Purcell et al., 2001)]. Combined, these approaches have been applied in previous studies to characterize the neurotypical neurobiological associates of the aforementioned neuroimaging features; as well as differences in these underpinnings that may lead to atypical neuroanatomy in autism. In the following, we will briefly outline the current state of this literature. Specifically, we will review general cortical development in neurotypicals and in autism; and the potential neurobiological correlates of individual features.

Broadly, the central nervous system develops from the neuroepithelium, which forms the neural plate and, later, the neural tube. At the start of neurogenesis [∼ gestational week four (Douet et al., 2014)], neuroepithelial cells generate a range of cells (including more neuroepithelial and radial glia progenitor cells) (Huttner and Kosodo, 2005). This generation of cells leads to a thickening of the cortical wall, giving rise to distinct zones (ventricular zone, subventricular zone, subplate/intermediate zone, cortical plate, and marginal zone) (Penisson et al., 2019). Prior to 6 weeks of gestational age, radial glia in the ventricular zone are thought to self-renew through symmetric cell division (amplification) (Penisson et al., 2019). At approximately 6 weeks of gestational age, however, radial glia transition to asymmetric division, resulting in an undifferentiated daughter stem cell (which undergoes further replication), and a progenitor cell that can divide and generate neurons in the subventricular zone (Noctor et al., 2001). At approximately 12–20 weeks (Volpe, 1981), according to the radial unit hypothesis (Rakic, 1978), neurons generated at the same site within the ventricular zone migrate along the radial glial progenitor cells (RGP) to form radial units (ontogenetic columns), with later generated neurons bypassing earlier generated neurons, creating an inside-out gradient of neurogenesis (Rakic, 1978). This developmental process is characteristic of pyramidal cells, which represent the majority (∼75–89%) of neurons in the cortex (Jones, 1984), and it is estimated that 87–90% of cells follow this path (Rakic, 1972). Other pathways exist, e.g., for interneurons, which follow a tangential migratory path to their target layer (Kriegstein and Noctor, 2004). However, these pathways are thought to have only minor effects on the development of cortical layers (Kriegstein and Noctor, 2004), and are beyond the scope of this review. This initial generation of a vast number of neurons is accompanied by gliogenesis (beginning at around gestational week 12) and followed by a period of both synaptogenesis and apoptosis [from around gestational week 20 until 4 years of age (Douet et al., 2014)], extensive arborization and myelination [both from gestational week 20 (Douet et al., 2014)], and pruning [from ∼12–25 years of age (Douet et al., 2014)]. In autism, disruptions in these processes can therefore result in numerous microstructural differences in the brain, that include ectopia/heterotopia, misoriented neurons, irregular lamination, increased neuron numbers, decreased neuron size, and other alterations (Wegiel et al., 2010; Chen et al., 2015). At the macroscopic level, these differences in turn give rise to atypical cortical thickness, surface area, cortical volume, lGI, and the GWC, as outlined in the following.

### 3.1. Cortical thickness

Cortical thickness, according to the radial unit hypothesis, is initially determined by the output of intermediate progenitor cells (i.e., the number of neurons) within each ontogenetic column (Rakic, 1978). Following an initial increase, and a peak at ∼1–2 years of age, cortical thickness declines progressively until adulthood (Huttenlocher, 1979; Fjell et al., 2015). This decline is thought to reflect extensive synaptic pruning, leading e.g., to a reduction in dendritic spine density and, subsequently, cell volume (Petanjek et al., 2011; Vidal-Pineiro et al., 2020). In autism, increased cortical thickness during childhood may e.g., result from excess neurons (possibly due to migration or pruning deficits), and a greater dendritic spine density and resulting number of synapses and neuronal size (Tang et al., 2014; Khundrakpam et al., 2017). In contrast, accelerated cortical thinning from adolescence onward has been suggested to result from an initial delay in synaptic/axonal pruning, which is thought to lead to a subsequent greater-than-normal neural loss (Lewis and Elman, 2008; Tang et al., 2014).

### 3.2. Surface area

Surface area, in contrast, is traditionally thought to be driven by the early amplification of radial glia, i.e., the amount of proliferative units and therefore the number of ontogenetic columns (Rakic, 1978), which likely correspond to the minicolumns observed in the adult brain (Krmpotić-Nemanić et al., 1984). Additionally, surface area may be influenced by, and influence other, cortical features. For instance, the expansion of the cortical sheet within the finite volume of the skull suggests a biological link between surface area and gyrification, although the direction of causality remains to be clarified. Surface area may also be influenced by factors that are inherent to the sMRI signal; for instance, as surface area is measured along the white matter surface, a blurring of the gray-white matter boundary may influence reliable surface area estimates (Storsve et al., 2014). Recently, emerging evidence also suggests that surface area measures may be related to the myelination of cortico-cortical axons (Cafiero et al., 2019). For instance, white matter growth may induce a tangential stretching of the brain, leading to an increase in surface area (Seldon, 2005). Alternatively, usage- or learning-dependent pruning (Bourgeois et al., 1994) and a proliferation of myelin into the neuropil (Sowell et al., 2003) have been proposed to influence surface area. Combined, these factors may lead to a rapid expansion of the cortex in childhood/adolescence, and a subsequent decline in the typically developing brain (Raznahan et al., 2011b; Hogstrom et al., 2013; Lyall et al., 2015). In autism, however, even minor disruptions to these crucial developmental processes, especially during the first 6 weeks of development, e.g., in the duration of divisions producing intermediate progenitor cells, may have profound impacts on the brain’s microstructure (White et al., 2010). Similarly, disturbances in white matter development [e.g., reviewed in Ecker (2017)] and synaptic pruning deficits (Tang et al., 2014) may contribute to the atypical expansion of the cortical surface in autism.

### 3.3. Cortical volume

Cortical volume is regulated through the interplay of the neurodevelopmental mechanisms that modulate its constituent components, namely cortical thickness and surface area. Hence, the neurobiological mechanisms affecting cortical volume development are complex, and inherently difficult to describe. Moreover, as mentioned above, the relative contributions of cortical thickness and surface area to cortical volume may vary across developmental stages; and may exert region-dependent effects (Im et al., 2008; Hazlett et al., 2011; Storsve et al., 2014). We therefore refer the reader to the mechanisms underpinning cortical thickness and surface area, which also affect measures of cortical volume, while highlighting the complex nature of this particular cortical feature.

### 3.4. Gyrification

The lGI captures the degree of regional folding (gyrification) of a certain brain area (or within a cortical patch). Gyrification, i.e., the formation of gyri and sulci, commences at 10–15 weeks of life, generating an adult-like pattern by the third trimester (Naidich et al., 1994). There are several explanations for cortical gyrification, which can be split broadly into two categories. First, tension-based or mechanical theories posit that gyrification results from the force of cortical fibers pulling together interconnected regions (e.g., Van Essen, 1997). Second, theories of differential tangential expansion suggest that cortical gyrification results from cortical layers/regions undergoing differential growth. Specifically, the difference in expansion of outer, relative to inner, layers may lead to a “buckling” and the generation of gyri and sulci (e.g., Retzius, 1891; Ronan et al., 2014). As mentioned above, gyrification and surface area (and cortical volume) are inherently linked as both need to be contained within the limited space of the skull. Hence, an initial increase in gyrification (White et al., 2010) may (at least partly) be driven by the processes that also help shape surface area and cortical volume (White et al., 2010). Similarly, the subsequent decline in gyrification during adulthood (Magnotta et al., 1999) has been linked to a concomitant accelerated decline in cortical volume during this time (Courchesne et al., 2011a), which may be driven by synaptic pruning (White et al., 2010). Accordingly, atypical gyrification in autism likely results from complex disruptions in the development of surface area, cortical volume, white-matter fibers (Ecker, 2017), neuronal generation and migration (Wegiel et al., 2010), and pruning (Tang et al., 2014). Nonetheless, further studies are needed to provide conclusive evidence on the developmental origins of the biological factors influencing the lGI in both the neurotypical and autistic brain.

### 3.5. Gray-white matter tissue contrast

The GWC is initially determined by the finely tuned patterning (acquisition of distinct cell identities) and spatial organization of cells and their components during development. In neurotypicals, fully migrated neurons in the cortical plate begin to elaborate dendritic and axonal elements from mid gestation through infancy (Haynes et al., 2005; Huang et al., 2006). These elements rapidly elongate, extending from the intermediate zone to intra- and subcortical targets to form synaptic connections (Haynes et al., 2005; Huang et al., 2006). The resulting groupings of somas (gray matter) and axons (white matter) possess different magnetic properties, which generate the signal contrast captured by sMRI. Accordingly, in the typically developing brain, changes e.g., in myelination and/or neuronal density across age may lead to a reduction in the GWC from childhood onward (Salat et al., 2009; Mann et al., 2018). In autism, increased GWC during childhood (Godel et al., 2021) and an atypical GWC developmental trajectory (in an inverted U-shape) during adolescence and adulthood (Mann et al., 2018) are likely to result from differences in either gray or white matter properties. This may include disruptions in the generation, differentiation, migration, and apoptosis of neurons, as well as the formation of axons. For instance, incomplete migration of intermediate progenitor cells into the cortical plate is thought to result in supernumerary neurons in the subplate and lead to a “blurring” of the gray-white matter tissue boundary (Bailey et al., 1998; Hutsler et al., 2007; Avino and Hutsler, 2010). Also, deficient apoptosis of subplate neurons that guide neurodevelopment may lead to greater retention of transient cells, further contributing to a blurrier boundary (McConnell et al., 1994). Taken together, variation in both gray and white matter may contribute to variation in the GWC; and further studies are required to dissect their relative contributions in the neurotypical and autistic brain.

## 4. Genetics

Surface-based neuroanatomical measures and their underlying (pathological) neurobiological processes are regulated through genetic and environmental factors, as well as gene-gene and gene-environment (epigenetics) interactions. Here, we will focus on genetic factors, which are thought to explain the majority of brain morphology [estimated heritability: 60–80% (Jansen et al., 2015)]. For reviews of environmental modulators and their interaction with genetic factors, see e.g (Tordjman et al., 2014; Hegarty et al., 2020).

Multiple commonly used statistical approaches exist to assess the genetic correlates of neuroanatomy and its associated biological processes. For example, single variant approaches examine genetic underpinnings (e.g., of a neuroanatomical feature) within a candidate gene framework focusing on functionally characterized polymorphisms (Bogdan et al., 2017) that are identified e.g., through Genome-Wide Association Studies (GWAS). Similarly, linkage studies can be used to identify loci that are associated with phenotypic traits within families (Dawn Teare and Barrett, 2005). Polygenic approaches be used to evaluate the additive effect of many genetic factors that may differ in their association with a trait. For instance, they include (1) polygenic risk scores (PRS), which summarize an individual’s genetic liability to a phenotype (e.g., a trait or a diagnostic label); and (2) biologically informed multilocus profile scores (BIMPS), which summarize polymorphisms across a given neural system to derive a composite of relative signaling within that pathway (Bogdan et al., 2017). To examine joint genetic variation within proteins/interactive networks in a hypothesis-driven or exploratory fashion, researchers may further use system- and pathway-level (enrichment) analyses (Bogdan et al., 2017). Finally, the additive effect of (these) genetic factors (A), common or shared environmental (C), and unique environmental factors and measurement errors (E) on phenotypes can be explored using multivariate approaches (e.g., ACE) (Douet et al., 2014). These approaches rely on various tools, including those that permit sequencing of the whole exome (all protein-coding genes), whole genome (including non-coding regions), single cells (genome and transcriptome of individual cells), and RNA (examines how each protein-coding gene is utilized in a given cellular context). Additionally, epigenetic tools exist that permit the quantification of DNA methylation (e.g., bisulfite sequencing), histone modification (e.g., chromatin immunoprecipitation), and chromosomal interactions that influence gene expression (e.g., chromosome conformation capture) (for a review, see e.g., Bae et al., 2015).

In combining these approaches, previous studies have explored the link between genetic variation and inter-individual variability in neuroanatomy indirectly (e.g., via the neurobiological mechanisms outlined above). Taken together, these studies have identified genetic and transcriptomic variation that converges in several key biological pathways, which may jointly influence cortical thickness, surface area, cortical volume, the local gyrification index, and the GWC. Further, emerging evidence, facilitated by progress in neuroimaging and statistical approaches, is examining the specific genetic factors contributing to neuroanatomical variability directly. In the following, we will provide an overview of the current state of research in both the neurotypical and autistic brain.

### 4.1. Genetic influences on neuroanatomy in neurotypicals

In the neurotypical brain, neuroanatomy and its development [beginning 2 weeks post-conception (Douet et al., 2014)] are largely regulated by genetic factors. This is highlighted by the fact that the reported heritability [i.e., the proportion of phenotypic (observable) variation in a trait that can be attributed to inherited genetic factors] of gray matter volume is greater than 50% overall [56% in neonates (Gilmore et al., 2010) and 82% in adults (Baaré et al., 2001), as summarized in Douet et al., 2014]. This heritability is thought to arise from the additive effect of hundreds of small-effect SNPs that are distributed across the entire genome (Toro et al., 2015). For example, GWAS studies have highlighted the relevance of (1) the COMT Va158Met polymorphism for cortical thinning from 9 to 22 years of age (Raznahan et al., 2011a), and (2) the 5-HTTLPR polymorphism in white matter microstructure in adolescents and adults (Pacheco et al., 2009). Also, previous studies have stressed sexual dimorphisms and the potential role of sex chromosomes in determining neuroanatomy and its development [e.g., reviewed in Douet et al., 2014]. Thus, while many findings await replication, emerging literature is implicating an increasing number of genes in the formation of brain structure.

Notably, the influence of these genes may vary across brain regions. For instance, a study in neurotypicals highlighted regional heterogeneity in the magnitude of genetic influence on cortical thickness (Rimol et al., 2010). Further, the role of these genes, and their expression in particular cell types, may change across age (Ball et al., 2020). This variability may be the result of various epigenetic factors, such as DNA methylation, histone modifications, microRNAs, and long noncoding RNAs (Douet et al., 2014). In fact, it is estimated that one third (i.e., 10,000 genes) of the whole genome is expressed solely during development (Johnson et al., 2009; Zhang et al., 2011); and that the rate of gene expression changes rapidly during fetal development, decelerates throughout childhood and adolescence, stabilizes in adulthood, and accelerates again after 50 years of age (Colantuoni et al., 2011). Moreover, gene expression patterns are thought to have a reverse relationship, whereby genes with the lowest expression during fetal development are highly expressed during aging and neurodegeneration and vice versa (Colantuoni et al., 2011; Douet et al., 2014). In sum, (the development of) neuroanatomy is tightly regulated through time-sensitive (epi)genetic influences. Consequently, neurodevelopmental conditions such as autism have been associated with alterations in the (epigenetic control of) genetic variation that contributes to neuroanatomy and its development.

### 4.2. Genetic influences on neuroanatomy in autism

In autism, differences in neuroanatomy have also been linked to genetic factors, although heritability estimates may differ from those in neurotypicals (Hegarty et al., 2020). Evidence for a potential genetic regulation of neuroanatomy in autism comes from a wealth of studies that have identified common and rare (epi)genetic variation in ∼1,000 genes (O’Roak et al., 2012). Combined, this variation may converge in the disruption of processes that are crucial to (the development of) neuroanatomical features. These processes include e.g., chromatin remodeling, Wnt signaling during development, and synaptic processes (De Rubeis et al., 2014; Krumm et al., 2014); as well as other pathways [e.g., disturbed protein synthesis and degradation, (mTOR-related) cell metabolism etc., reviewed in Bourgeron, 2015 and de la Torre-Ubieta et al., 2016] that are beyond the scope of this review. Similarly, for a comprehensive study of the genetic effect on neuroanatomy of multiple genetic mouse models of autism, we refer the reader to previous work (e.g., Ellegood et al., 2015).

Chromatin modification can influence early neuroanatomical development, e.g., by altering the expression of genes regulating the fate of neuronal progenitor cells [proliferation, neuronal lineage commitment and differentiation (Lasalle, 2013; Cotney et al., 2015)]. Consequently, its disturbance may affect the establishment of neurotypical neuroanatomy. In autism, examples of reported chromatin-related anomalies include mutations in genes encoding chromatin modifiers and regulators and transcription factors, such as MECP2, CHD8, ARID1B, CBX4, KDM6B, MLL3/5, SMARCC2, SETD2, MEF2A etc (O’Roak et al., 2012; Ben-David and Shifman, 2013; Parikshak et al., 2013; Bourgeron, 2015; Sahin and Sur, 2015). Other examples include genetic variants that are located outside of protein coding exons, but influence the binding and actions of chromatin factors and DNA methylation patterns (Lasalle, 2013). Wnt signaling may help shape neuroanatomy e.g., by regulating radial glia self-renewal, neurogenesis, and neuronal differentiation (Hansen et al., 2011; Tiberi et al., 2012). Accordingly, overexpression of β-catenin, a key component of the Wnt canonical pathway, has been reported to lead to a drastic expansion of cortical surface area and thinning of the cortex (Chenn and Walsh, 2002; Wrobel et al., 2007). In autism, genetic variation associated with Wnt signaling includes, e.g., CTNNB1, which encodes β-catenin (Krumm et al., 2014); DLL1, which is expressed in neural progenitor cells (Barton and Fendrik, 2013); and TBL1XR1, which binds β-catenin (Cadigan, 2008). Last, previous studies in autism have highlighted synaptic genes (Voineagu et al., 2011; de la Torre-Ubieta et al., 2016), which may affect neuroanatomy e.g., by altering dendritic arborization and spine density. In autism, atypical synaptic genes include those associated with synaptic cell adhesion molecules like neurexins [e.g., NRXN2 and NRXN3 (Wang et al., 2018)] and neuroligins [e.g., NLGN3, NLGN4 (Jamain et al., 2003)]; synaptic scaffolding molecules [e.g., SHANK3 (De Rubeis et al., 2014)]; synaptic receptors and transmitters [e.g., GABRB3 (Buxbaum et al., 2002), GluR6 (Jamain et al., 2002)], and HTT (locus SLC6A4), which encodes the serotonin transporter (Tordjman et al., 2001); and ion channels [e.g., SCN2A, encoding NAV1.2 channels; CACNA1D, encoding the CAV1.3 channel; and CACNA2D3, encoding the α2δ-3 subunit (De Rubeis et al., 2014)].

Notably, these processes are heavily interlinked (epistasis). For example, chromatin-related genes, such as CHD8, are thought to help shape regulatory expression networks in the developing brain; and to influence other autism risk/liability genes (Cotney et al., 2015), such as p53, a regulator of apoptosis (Nishiyama et al., 2009), and the Wnt/β-catenin target genes (Lasalle, 2013). This underscores the pleiotropy of many autism genes, whereby variations in certain genes (some of which appear to affect other genes) result in a wide range of phenotypic expressions (Lee et al., 2021). Conversely, many autism-related genetic anomalies also converge onto common pathways that jointly affect cortical development, chromatin modification, Wnt signaling, and synaptic functioning (Hashem et al., 2020), and therefore influence neuroanatomical features like cortical thickness and surface area (van der Meer et al., 2020). Further, genetic influences on neuroanatomy may be region-specific. For instance, previous studies suggest that physically adjacent brain regions display positive genetic correlations (e.g., for cortical thickness and surface area), while more distal regions are negatively correlated (Grasby et al., 2020). Also, the penetrance and phenotypic expression of genetic variants may vary, e.g., as reported in probands with copy number variants in NRXN1, which showed no clinically overt brain-related phenotype (Woodbury-Smith et al., 2017).

In sum, neuroanatomy has been associated with a wealth of genomic factors that may further interact; and whose disruptions (e.g., in autism) may give rise to atypical neuroanatomy and neuroanatomical development. In the following, we will briefly review the existing literature on how genomic factors may influence individual cortical features in the neurotypical and atypical (autistic) brain.

### 4.3. Genetic influences on cortical thickness

In neurotypicals, estimates of how much variance in cortical thickness is determined by genetic factors vary across studies [e.g., average cortical thickness: 26% (Grasby et al., 2020); regional cortical thickness: 13% (Grasby et al., 2020); heritability: 69–81% (Panizzon et al., 2009; Winkler et al., 2010)]. Nonetheless, several studies have associated neuroanatomical variability in cortical thickness with common and rare variants. For instance, in a large-scale study including participants ranging from childhood to old age, average cortical thickness was linked to RPSA, which is associated with laminin, a regulator of neurogenesis, neuronal differentiation, and neuronal migration (Solozobova et al., 2012; DiGiacomo and Meruelo, 2016); and ACTR1B, which helps modulate the dynactin complex involved in neuronal migration (Itoh, 2016). Notably, variability in cortical thickness was enriched for active regulatory elements (promoters and enhancers) that are adult-specific and influence processes such as myelination, pruning, and branching (Grasby et al., 2020). Evidence from a large-scale GWAS (Hofer et al., 2020) has further linked regional variability in cortical thickness to variation in pathways associated with autism, including Wnt signaling (e.g., WNT3, DAAM1) and transcription (e.g., SALL1). Further, studies in individuals ranging from childhood to old age reported that cortical thinning was associated with interregional patterns not only of neuronal, but also of glial gene expression (Shin et al., 2018; Vidal-Pineiro et al., 2020). Neuronal gene expression included markers of CA1 pyramidal cells, e.g., ADGRB3, involved in synaptic functioning (Sticco et al., 2021); CDH2, implicated in neuronal differentiation (Alimperti and Andreadis, 2015); and CHL1, aiding neuronal survival (Chen et al., 1999). Glial gene expression comprised genes associated with microglia, e.g., ALOX5, involved in synaptic function (Joshi et al., 2014); and BLNK and CYBB, both implicated in immune function (Rae et al., 1998; Minegishi et al., 1999). It also included astrocytic genes, such as ALDH5A1, an indirect regulator of GABA catabolism (DiBacco et al., 2020); and ASTN1, a promoter of neuronal migration (Horn et al., 2018). Less thinning was associated with greater gene expression in development. Moreover, this relationship was reversed during aging (Vidal-Pineiro et al., 2020). This further highlights the temporal specificity of the genetic regulation of neuroanatomy. Moreover, in neurotypical children, greater cortical thickness was associated with greater polygenic scores for autism (Grove et al., 2019; Khundrakpam et al., 2020). Studies of rare variants further reported that, in neurotypical children and adolescents, reduced cortical thickness in “social brain” regions, and greater autism symptomatology [increasing scores on the Social Responsiveness Scale (SRS) (Constantino et al., 2003)], were associated with greater variation (G->C allele) in the MET receptor tyrosine kinase promotor (Campbell et al., 2006; Hedrick et al., 2012). MET is thought to help regulate synaptogenesis, and migration and is also an autism risk gene (Campbell et al., 2006; Hedrick et al., 2012). In line with this, we have previously reported that variation in cortical thickness (development) in brain regions associated with autism (core) symptoms was genetically enriched for a range of genes implicated in autism, including those involved in (excitatory) synaptic transmission and development (Bieneck et al., 2021; Ecker et al., 2022). Combined, these studies further underscore a link between atypical neuroanatomy and genetic disruptions, even in the absence of a clinically manifested diagnosis Table 1.

**TABLE 1:** The genetic influences on neuroanatomy in neurotypicals (NT genes) and individuals with autism (Autism genes), and their functional roles, that are mentioned in this review.

NT genes	Association	References	Autism genes	Association	References
**CT**					
RPSA	Laminin, neurogenesis, neuronal differentiation, and neuronal migration	DiGiacomo and Meruelo, 2016	SCN1A	Voltage-gated sodium channels	Weiss et al., 2003
ACTR1B	Modulates the dynactin complex involved in neuronal migration	Itoh, 2016	SLIT1, SLIT3	Axonal midline crossing	Plump et al., 2002
WNT3	Wnt signalling	Hofer et al., 2020	GABRA3, GABRA5	GABA receptor subunit	Parikshak et al., 2016
DAAM1	Wnt signalling	Hofer et al., 2020	GABRB1	GABA receptor subunit	Parikshak et al., 2016
SALL1	Transcription	Hofer et al., 2020	PTCHD1	Excitatory synapses	Ross et al., 2020
ADGRB3	Synaptic functioning	Sticco et al., 2021	SYN2	Synapsin 2	Corradi et al., 2014
CDH2	Neuronal differentiation	Alimperti and Andreadis, 2015	SYT17	Synaptotagmin 17	Ruhl et al., 2019
CHL1	Neuronal survival	Chen et al., 1999	FMR1	Fragile X messenger ribonucleoprotein	DiMario, 2004
ALOX5	Microglia, synaptic functioning	Joshi et al., 2014	TSC1	Neuronal growth and migration	Fu et al., 2012
BLNK	Immune function	Minegishi et al., 1999			
CYBB	Immune function	Rae et al., 1998			
ALDH5A1	GABA catabolism	DiBacco et al., 2020			
ASTN1	Neuronal migration	Horn et al., 2018			
MET	Synaptogenesis, migration	Campbell et al., 2006; Hedrick et al., 2012			
**SA**					
INA	Neuronal migration	Baum and Garriga, 1997	BDNF	Neuronal proliferation and plasticity	Raznahan et al., 2009
AS3MT	Neural plasticity	Zhao W. et al., 2021			
NT5C2	Neural progenitor cells	Duarte et al., 2019			
EIF2AK4	Synaptic plasticity, neuritogenesis	Roffé et al., 2013			
THBS1	Synaptogenesis	Jayakumar et al., 2014			
ST7L	Wnt pathway	Savage et al., 2018			
WNT2B	Wnt pathway	Savage et al., 2018			
NSF	Synaptic transmission	Nishimune et al., 1998			
RSPO3	Wnt signalling	Katoh and Katoh, 2017			
FOXO3	Cell survival and apoptosis	Pino et al., 2014			
**CV**					
IGFBP7	Learning, memory, neurogenesis	Xia et al., 2017	SLC6A4	Serotonin transporter gene	Katoh and Katoh, 2017
ZNF804A	Zinc finger protein	Wei et al., 2015	BDNF	Brain-derived neurotrophic factor	Baum and Garriga, 1997
DAAM1	Histone modification	van der Lee et al., 2019; Hofer et al., 2020	OXTR	Oxytocin receptor	Pino et al., 2014
THBS3	Histone modification	van der Lee et al., 2019			
ZIC4	Transcriptional regulation	van der Lee et al., 2019			
FGFRL1	Fibroblast growth factor receptor	van der Lee et al., 2019			
RSPO3	Wnt signalling	Hofer et al., 2020			
NSF	Synaptic transmission	Hofer et al., 2020			
FOXO3	Cell survival and apoptosis	Hofer et al., 2020			
**Gyrification**					
SEMA3A	Axon guidance	Vo et al., 2013	MECP2	Rett syndrome	Keidar et al., 2019
ROBO2	Neuronal cell differentiation	Sung et al., 2013	LIS1	Lissencephaly	Keidar et al., 2019
NAV2	Neuronal migration	Pook et al., 2020; van der Meer et al., 2021			
**Gyrification**					
SEMA3A	Axon guidance	Vo et al., 2013	MECP2	Rett syndrome	Keidar et al., 2019
ROBO2	Neuronal cell differentiation	Sung et al., 2013	LIS1	Lissencephaly	Keidar et al., 2019
NAV2	Neuronal migration	Pook et al., 2020; van der Meer et al., 2021			
**GWMC**					
MIR137 (RNA gene)	Development, differentiation	The Schizophrenia Psychiatric Genome-Wide Association Study [GWAS] Consortium, 2011			
CACNAC1C	Calcium channel signaling	The Schizophrenia Psychiatric Genome-Wide Association Study [GWAS] Consortium, 2011			
ANK3	Cell activation, proliferation	The Schizophrenia Psychiatric Genome-Wide Association Study [GWAS] Consortium, 2011			
ITIH3-ITIH4		The Schizophrenia Psychiatric Genome-Wide Association Study [GWAS] Consortium, 2011			
VCAN	Early cell remodeling	Wathlet et al., 2011			
NBEAL1	Fetal development	Chen et al., 2004			
CACNB2	Cortical thickness	Chen et al., 2020			

Please note that this list is not comprehensive. For comprehensive reviews of genetic factors implicated in autism, see e.g (Woodbury-Smith and Scherer, 2018; Wiśniowiecka-Kowalnik and Nowakowska, 2019). CT, cortical thickness; CV, cortical volume; GWMC, grey-white matter tissue contrast; SA, surface area. Findings highlighted in grey did not replicate in follow-up studies (Papiol et al., 2014).

In autism, recent progress in the availability of large-scale datasets is now enabling research into how atypical cortical thickness (development) is influenced by common genetic variation. For example, in autistic children, global cortical thickness differences were enriched for variation in genes downregulated in autism (Parikshak et al., 2016). Among others, these comprised genes involved in neurodevelopment and the regulation of synaptic processes. They included SCN1A, which encodes voltage-gated sodium channels (Weiss et al., 2003); SLIT1 and SLIT3, which help regulate axonal midline crossing in the developing brain (Plump et al., 2002); GABRA3, GABRA5, and GABRB1, which encode GABA receptor subunits (Parikshak et al., 2016); PTCHD1, which helps regulate excitatory synapses (Ross et al., 2020); and SYN2 and SYT17, which encode synapsin 2 and synaptotagmin 17, respectively, and regulate synaptic functioning (Corradi et al., 2014; Ruhl et al., 2019). Also, a recent study in children, adolescents, and adults reported that deviations in cortical thickness from the neurotypical developmental trajectory were associated with polygenic risk for autism (Ecker et al., 2022). Moreover, regional differences in cortical thickness were enriched for genes (both common and rare variants) previously associated with autism, including those involved in cell differentiation, synaptic functioning, transmembrane receptor activity, synaptic transmission, cell adhesion signaling, and nuclear functioning (Voineagu et al., 2011; Parikshak et al., 2016; Gandal et al., 2018). Regions where autism subgroups differed in sensory processing were further enriched for genes linked to the (migration of) excitatory neurons, including in deep layer 1 and 2 (Polioudakis et al., 2019; Ecker et al., 2022); highlighting potential within-group variability in the relationship between neuroanatomy and genetic variation in autism. Previous research has also examined how cortical thickness is impacted by rare genetic variants. For instance, studies have identified atypical cortical thickness in common single-gene conditions associated with autism, such as Fragile X Syndrome (Shelton et al., 2016); where atypical methylation of FMR1 contributes to disruptions in brain development; and Tuberous sclerosis (DiMario, 2004), where variation e.g., in TSC1 has been linked to a disruption in neuronal growth and migration (Fu et al., 2012). Combined, these studies provide strong evidence for the notion that inter-individual variation in genes (including those involved in biologically plausible pathways implicated in autism) is linked to atypical cortical thickness (development) in autism Table 1.

### 4.4. Genetic influences on surface area

In the typically developing brain, surface area is thought to be determined primarily by genetic factors, although heritability estimates vary across studies [e.g., 71–89% (Panizzon et al., 2009; Winkler et al., 2010)]. Among these genetic determinants, common genetic variants may explain up to 34% of variance in total, and 8–31% in regional, surface area (Grasby et al., 2020). In line with this, a recent large-scale study has linked total surface area variability to multiple genetic variants, such as INA, which is associated with neuronal migration (Baum and Garriga, 1997); AS3MT, related to neural plasticity (Zhao W. et al., 2021); and NT5C2, linked to signaling in neural progenitor cells (Duarte et al., 2019). The same study linked regional surface area variability to variants including EIF2AK4, which is associated with synaptic plasticity and neuritogenesis (Roffé et al., 2013); THBS1, a modulator of synaptogenesis (Jayakumar et al., 2014), and many single nucleotide polymorphisms (SNPs) related to the Wnt pathway, such as ST7L and WNT2B (Savage et al., 2018). Similarly, evidence from a large-scale GWAS (Hofer et al., 2020) has linked both global and regional surface area variability to variation in genes involved synaptic transmission (e.g., NSF) (Nishimune et al., 1998), Wnt signaling (e.g., RSPO3) (Katoh and Katoh, 2017), and cell survival and apoptosis (e.g., FOXO3) (Pino et al., 2014). Notably, variation in surface was associated with genetic variants that alter regulatory activity in neural progenitor cells during fetal development (unlike cortical thickness, where enrichment was adult-specific) (Grasby et al., 2020). Combined, this suggests that both total and regional surface area are influenced by (time-sensitive) genetic variation in pathways that contribute to the phenotype of surface area; and that atypical surface area may therefore reflect variation in these genetic correlates Table 1.

In autism, surface area is also thought to be largely heritable (Hegarty et al., 2020), but little is known about the specific genetic contributions to this morphometric measure. Among the relatively few studies examining the relationship between surface area and genetic variation in autism (compared to neurotypicals), a previous study in children, adolescents, and adults reported that frontal surface area and cortical volume were regulated differently by a single nucleotide polymorphism in BDNF (BDNF Val66met genotype) (Raznahan et al., 2009). BDNF helps regulate neuronal proliferation and plasticity (Raznahan et al., 2009), and may therefore plausibly influence surface area. Further, we have previously shown that in autism, surface area in brain regions associated with variation in adaptive outcome were enriched for (1) genes commonly downregulated in autism (Gandal et al., 2018) (OR = 3.07), (2) co-expression modules downregulated in autism (Parikshak et al., 2016), and (3) genes differentially expressed in bipolar disorder and schizophrenia (Gandal et al., 2018; Pretzsch et al., 2022). Last, altered surface area has also been identified in conditions genetically and clinically associated with autism, such as 16p11.2 deletion and duplication syndromes (Qureshi et al., 2014). Combined, these studies suggest that there may be clinical/biological subgroups in autism which display distinct relationships between (altered) neuroanatomy and genetic variation Table 1.

### 4.5. Genetic influences on cortical volume

In neurotypicals, cortical volume (like its components cortical thickness and surface area) is thought to be determined largely (up to ∼90%) by genetic factors (Baaré et al., 2001; Wallace et al., 2006). For instance, in neurotypical infants, brain volume has been shown to be associated with variation in IGFBP7, which may be linked to learning, memory, and neurogenesis (Xia et al., 2017). Moreover, in adults, cortical volume (and surface area) in frontal, precentral, and parietal regions was linked to variation in the ZNF804A gene (Wei et al., 2015). A recent large-scale study further suggests that the genetic influence on cortical volume may differ across regions; in fact, volumes of individual lobes were associated with specific genetic variants located in regions enriched for histone modifications (DAAM1 and THBS3) and close to genes causing Mendelian brain-related diseases (ZIC4 and FGFRL1) (van der Lee et al., 2019). Given that cortical volume is the product of cortical thickness and surface area, it is unsurprising that the genetic correlates of these features (e.g., DAAM1, RSPO3, NSF, and FOXO3) overlap (Hofer et al., 2020). In sum, the existing research indicates that the link between cortical volume and genetic variation may be region-specific (and potentially age-dependent); and additional research is required to elucidate the mechanisms that regulate these relationships Table 1.

In autism, cortical volume is thought to be determined largely by genetic factors (Hegarty et al., 2020); but few studies have identified individual rare, let alone common, genetic contributors to cortical volume in autistic people. In children, adolescents, and adults, we have previously reported that cortical volume differences in clinical outcome-related regions were enriched for (common and rare) gene sets that are downregulated in autism (Gandal et al., 2018) and co-expression modules upregulated in autism (Parikshak et al., 2016). Also, in autistic children, atypical cortical gray matter volume has been associated with 5-HTTLPR, a promoter of the serotonin transporter gene SLC6A4 (Wassink et al., 2007). A study in adolescents and adults found that frontal cortical volume was regulated atypically in the BDNF val66 met genotype (Raznahan et al., 2009). In autistic adults, atypical cortical volume in the insula was linked to variation in OXTR, the oxytocin receptor gene (Saito et al., 2014). Combined, these studies provide preliminary evidence that cortical volume disruptions may be related to genetic variation in (biologically plausible) pathways that influence the cortical volume phenotype; but little is known about the (distinct) cortical thickness- and surface area-linked genetic and biological contributors to this relationship Table 1.

### 4.6. Genetic influences on gyrification

In neurotypicals, gyrification (unlike other morphological features) has been reported to be determined largely by non-genetic factors (Hegarty et al., 2020; Schmitt et al., 2021); and the (limited) influence of genetic factors on gyrification may vary across the brain (Pizzagalli et al., 2020; Schmitt et al., 2021). Further, a recent study in neurotypical adolescents and adults, has linked cortical folding to patterns of local genetic correlations in cortical thickness. More specifically, the authors suggested that cortical folding may be due to patterned local influences on cortical thickness that may reflect e.g., molecular signaling gradients and cellular variation (Alexander-Bloch et al., 2020). Similarly, a recent study (van der Meer et al., 2021) highlighted significant genetic overlap between gyrification (sulcal depth), cortical thickness, and surface area. The study also identified multiple genetic loci linked directly to cortical folding (sulcal depth), including e.g., SEMA3A, which is involved in axon guidance (Vo et al., 2013); ROBO2, linked to (neuronal) cell differentiation (Sung et al., 2013); and NAV2, which is implicated in neuronal migration (Pook et al., 2020; van der Meer et al., 2021). Combined, these studies suggest that gyrification may be genetically/biologically coupled with other cortical features; but the distinct genetic contributors to each feature, and their relationships with each other, remain unclear Table 1.

In autism, similar to neurotypicals, heritability studies suggest that gyrification is only minorly influenced by genetic factors (Kates et al., 2009); and research into these factors remains scarce to non-existent. For instance, preclinical research studies suggest that MECP2, the main causative gene of Rett syndrome, which is also associated with autism, interacts on a molecular level with LIS1, the main causative gene for lissencephaly (Keidar et al., 2019); suggesting a potential genetic association between gyrification and autism. However, these findings remain to be validated in humans. Moreover, as gyrification is associated with cortical thickness and surface area, it is likely that the genetic factors modulating these features also influence gyrification. For example, according to the tension based on model of gyrification, it is possible that atypical gyrification in autism is (at least partly) modulated by genetic differences in white matter (for a review, see Travers et al., 2012). However, additional research is crucial to identify the genetic influences on cortical gyrification, both in the neurotypical and autistic brain Table 1.

### 4.7. Genetic influences on gray-white matter tissue contrast

In neurotypical adults, the GWC is thought to be determined (in part) by regionally specific genetic influences [heritability 0.24–0.5 (Panizzon et al., 2012)]. As evident in the name, the contrast depends on the properties of both gray and white matter. However, a recent study in neurotypical adults found no genetic correlation between the GWC and the gray-matter based cortical thickness, suggesting separate sources of genetic variance for these two features (Panizzon et al., 2012); while the genetic correlations between the GWC and surface area remain to be investigated. Similarly, the genetic link between the GWC and white matter remain poorly understood. Previous studies suggest that white matter may be regulated through a complex set of genes, such as MIR137, CACNA1C, ANK3, and the ITIH3-ITIH4 region (The Schizophrenia Psychiatric Genome-Wide Association Study [GWAS] Consortium, 2011); but efforts to replicate these specific findings have failed (Papiol et al., 2014). Other GWAS (Zhao B. et al., 2021) have identified additional (to be validated) genetic variants underpinning white matter, including VCAN, implicated in early cell remodeling (Wathlet et al., 2011); NBEAL1, implicated in fetal development (Chen et al., 2004); and CACNB2, which has been associated with cortical thickness in bipolar disorder (Chen et al., 2020). Moreover, in neurotypical children, white matter connectivity between frontal and parietal regions was associated with higher autism polygenic scores (Grove et al., 2019; Khundrakpam et al., 2020). Notably, a recent study in eight psychiatric/mental health conditions found that the white matter associated genetic variant DCC (a regulator of white matter projections/axonal guidance during development) was the most highly pleiotropic locus across conditions (Lee et al., 2021). Combined, these studies highlight the biological and genetic complexity of the GWC; and the (potentially) low specificity and high pleiotropy of the implicated genetic variants Table 1.

In autism, the genetic variation influencing atypical GWC remains largely unstudied. Studies in autistic children and adolescents have linked atypical white matter (volume) to mutations in PTEN, which has been associated with neural connectivity, plasticity, and apoptosis (van Diepen and Eickholt, 2008; Frazier et al., 2015). However, additional research is required to examine the genetic correlates of the GWC in this condition.

## 5. Current and future trends

Taken together, research into the biological and genetic underpinnings of neuroanatomy is rapidly expanding. This work has been facilitated by the emergence of new fields of research (e.g., imaging genomics, which aims to link phenotypic features to associated genomic variation); and novel statistical and technological approaches [e.g., linkage disequilibrium (LD) score based regression, which permits the genetic correlation of different phenotypes while controlling for intrinsic correlation existing between genetic loci in LD blocks (Bulik-Sullivan et al., 2015)]. Combined, this progress has enhanced both data-driven exploratory studies, e.g., aimed at identifying new aetiological mechanisms/variants; and hypothesis-driven research, e.g., seeking to confirm previously implicated neurobiological features. Nonetheless, several limitations remain to be addressed to realize the potential of these approaches. These include frequently restrictive sample sizes, which reduce the power of studies to detect statistically significant effects. For instance, just under 200 participants are required to detect an effect size of 80% at *p* = 0.01 (in an independent sample *t*-test), not yet accounting for multiple comparison corrections (Carter et al., 2017). Studies aiming to associate inter-individual differences in brain structure or function with complex cognitive or mental health phenotypes (i.e., brain-wide association studies) may even require thousands of individuals to be reproducible (Marek et al., 2022). These sufficiently large sample sizes are particularly crucial in view of the aetiological and phenotypic heterogeneity in autism and the multitude of examined features (e.g., brain regions or genetic loci).

Another limitation is that replication efforts are frequently hampered (e.g., by practical considerations, such as the lack of an appropriate independent dataset and associated costs) and are therefore often limited to within-sample cross validation etc. Fortunately, several research collaborations and consortia [e.g., EU-AIMS (European Autism Interventions — A Multicenter Study for Developing New Medications) and AIMS-2-TRIALs (Autism Innovative Medicine Studies-2-Trials) (Murphy and Spooren, 2012), ENIGMA (Enhancing NeuroImaging Genetics through Meta Analysis) (Thompson et al., 2014), the autism genome project (Hu-Lince et al., 2005), R2D2 (Risk and Resilience in Developmental Diversity and Mental Health) with a link to the website: www.r2d2-mh.eu the autism genetic resource exchange (Geschwind et al., 2001), and the UK biobank (Ollier et al., 2005)] are currently underway to tackle these challenges in increasingly large and heterogeneous participant groups. Harnessing technical advances, such as individual-level statistics (e.g., normative modeling approaches) or (cross-modal) clustering techniques (e.g., canonical correlation analysis and principal independent component analysis), these studies are increasingly equipped to parse the aetiological/pathophysiological heterogeneity of autism, and to put new findings into rapidly updated frameworks of pathology. Further, by including diverse groups of participants across age, these studies also permit glimpses into the specificity and commonality of (e.g., aetiological) features to autism and related (co-occurring) conditions. For instance, a recent study in more than 200 children reported neuroanatomical differences in autism that were shared by individuals with attention-deficit/hyperactivity disorder (Kushki et al., 2019).

Combined, this research is crucial not only to elucidate biological mechanisms in autism, but also to help inform clinical practice. For example, the stratification of autistic individuals into neurobiologically homogeneous subgroups may aid the development of interventions tailored to these subgroups (“precision medicine”); for a recent “white paper” regarding the use of biomarkers in precision medicine, (see Beversdorf, 2016). Further, linking imaging features and associated biological mechanisms to clinical outcome may boost future diagnostic processes, which are currently restricted to an assessment of behavior. For example, we have found preliminary evidence that individual- and group-level neuroanatomical variability predict longitudinal clinical outcome associated with core features in autism (Pretzsch et al., 2022). Once validated, such insights into (individual-level) genetic and pathophysiological mechanisms underpinning clinical profiles could provide a basis for (supplementary) diagnostic tools.

## 6. Conclusion

In this review, we have discussed commonly studied neuroanatomical features and their potential biological mechanistic and genetic underpinnings in the neurotypical and autistic brain. Most of the reviewed studies focused on cortical thickness and surface area; while research into other features (lGI, GWC) and their neurobiological correlates was scarce. Combined, the reviewed literature suggests that neuroanatomical features possess both distinct and shared neurobiological contributors whose variability between regions and across age is regulated dynamically by a complex interplay of epigenetic factors. Examples of distinct contributors include the number of proliferative units (which determines surface area) compared to the number of cells in each unit (which determines cortical thickness) [see radial unit hypothesis (Rakic, 1978)]. However, these contributors may originate from shared pathways, e.g., the development of progenitor cells; and are likely polygenic, i.e., influenced by more than one gene. Shared neurobiological contributors include, e.g., the association of surface area with both cortical volume and gyrification; and the genetic overlap between cortical thickness and surface area (Jha et al., 2018), which underscores the pleiotropy of many (autism) genes. Notably, this aetiological and pathophysiological overlap between imaging features may affect their suitability as an intermediate phenotype to examine underpinning mechanisms in autism. To address this, it is crucial to clearly demarcate the investigated feature; and to chart a potential mechanistic pathway from imaging feature through neurobiological mechanisms to genomic factors to contextualize and interpret findings appropriately. Such efforts are currently being facilitated by several large-scale research collaborations examining large and heterogeneous samples. Combined, these studies may not only improve our understanding of biological mechanisms in autism, but also help stratify individuals to support the development of targeted clinical interventions.

## Author contributions

Both authors were involved in the conceptualization, writing, draft preparation, review and editing of the manuscript and read and agreed to the published version of the manuscript.
